# Upregulated Expression of TUBA1C Predicts Poor Prognosis and Promotes Oncogenesis in Pancreatic Ductal Adenocarcinoma via Regulating the Cell Cycle

**DOI:** 10.3389/fonc.2020.00049

**Published:** 2020-02-14

**Authors:** Mugahed Abdullah Hasan Albahde, Piao Zhang, Qiuqiang Zhang, Guoqi Li, Weilin Wang

**Affiliations:** ^1^Department of Hepatobiliary and Pancreatic Surgery, School of Medicine, The Second Affiliated Hospital, Zhejiang University, Hangzhou, China; ^2^Key Laboratory of Precision Diagnosis and Treatment for Hepatobiliary and Pancreatic Tumor of Zhejiang Province, Hangzhou, China; ^3^Research Center of Diagnosis and Treatment Technology for Hepatocellular Carcinoma of Zhejiang Province, Hangzhou, China; ^4^Clinical Medicine Innovation Center of Precision Diagnosis and Treatment for Hepatobiliary and Pancreatic Disease, Zhejiang University, Hangzhou, China; ^5^Department of Anesthesiology, School of Medicine, Sir Run Run Shaw Hospital, Zhejiang University, Hangzhou, China; ^6^Clinical Research Center of Hepatobiliary and Pancreatic Diseases of Zhejiang Province, Hangzhou, China

**Keywords:** TUBA1C, pancreatic ductal adenocarcinoma, prognosis, proliferation, cell cycle

## Abstract

**Background:** Pancreatic ductal adenocarcinoma (PDAC) is a highly malignant disease and has the worst prognosis and survival rate. TUBA1C is a microtubule component implicated in multiple cancers, however, the clinical significance and biological functions of TUBA1C in the progression of PDAC remain unexplored.

**Methods:** The Cancer Genome Atlas (TCGA) and Gene Expression Profiling Interactive Analysis (GEPIA) data were employed to detect the TUBA1C mRNA expression and the relation between TUBA1C expression and overall survival (OS) in PDAC. Then, bioinformatic analysis was employed to determine the potential pathway and genes related to TUBA1C. Human pancreatic cancer tissue and adjacent non-tumor tissues samples were detected by immunochemistry (IHC) staining, and the correlation between TUBA1C expression and the clinicopathological features were investigated. Meanwhile, TUBA1C expression in PDAC cell lines was evaluated by western blotting. Furthermore, functional assays including cell viability, apoptosis, cell cycle, transwell assay, wound healing assay, and a xenograft tumor model were performed to determine the oncogenic role of TUBA1C in PDAC, respectively.

**Results:** TUBA1C was overexpressed in the PDAC tissues and cells. IHC analysis showed that the TUBA1C overexpression was associated with short OS. Bioinformatic analysis indicated that TUBA1C overexpression was mainly associated with cell cycle regulation. The downregulation of TUBA1C significantly suppressed cell proliferation, induced cell apoptosis and cycle arrest, and inhibited invasion and migration in PDAC cells. Furthermore, TUBA1C downregulation also inhibited tumor growth *in vivo*.

**Conclusion:** These findings suggested that TUBA1C downregulation suppressed PDAC aggressiveness *via* cell cycle pathway and that TUBA1C may serve as a potential prognostic marker for PDAC therapy.

## Introduction

Pancreatic ductal adenocarcinoma (PDAC) is one of the most lethal cancers with a 5-year survival rate of <8% worldwide (1, 2). The morbidity and mortality of PDAC were gradually increased according to the survey data (3). Currently, surgery is the only radical cure for patients with PDAC and provides the only chance of long-term survival by improving the 5-year survival rate (4). However, it is difficult to diagnose at a curable stage because of the absence of apparent clinical signs or symptoms and the lack of sensitive and specific markers (5, 6). Therefore, surgical resection is not applicable for over 80% of patients in which the disease is locally advanced or metastasized (7). For patients with metastatic disease, surgery endures underuse, and systemic chemotherapy is the leading choice. At present, chemotherapy, including 5-fluorouracil (5-FU), gemcitabine, erlotinib, FOLFIRINOX, paclitaxel, capecitabine, and irinotecan have been widely applied in clinics. But the advances in chemotherapy for PDAC have been limited in comparison with other cancers owing to the significant drug resistance (8). Consequently, it is critical to clarify the molecular mechanism of PDAC and exploit useful specific biomarkers to predict the prognosis and guide individualized therapy in patients with PDAC.

Microtubules are vital components of the cytoskeleton and one of the most multifunctional proteins involved in a dynamic process of polymerization and depolymerization through cell replication and division (9). Microtubules are formed of α-tubulin and β-tubulin heterodimers (of dimensions 4 × 5 × 8 nm and 100,000 Da in mass) organized in the form of slender filamentous tubes (10, 11). The researches exposed that microtubules combined with many microtubule-related proteins aggregate to achieve various cellular functions such as maintenance of cell morphology, intracellular material transport, secretion of vesicles, cell deformation, movement, and division and chromosome separation in the cell cycle (mitosis and meiosis) (12). Moreover, microtubules play critical functions in regulating the mitotic apparatus to affect cell proliferation, differentiation, and apoptosis (12, 13). Recently, numerous researches showed that several tubulin isotypes including α-, β-, and γ-tubulin were significantly implicated in the progression of tumors including small-cell lung cancer, thymic epithelial tumor, breast cancer, gastric cancers, and renal cell carcinoma (14–18). Particularly, α-tubulin, as one of the microtubule components, plays pivotal roles in various cancers (19). Moreover, TUBA1C, a subtype of α-tubulin, which is composed of microtubule structure, was reported to be overexpressed and predicts poor prognosis and promotes cell proliferation and migration in hepatocellular carcinoma (HCC) (20, 21). However, the relevant effect of TUBA1C in PDAC patients has not been reported. Accordingly, we attempted to further evaluate the expression of TUBA1C and its associations with clinicopathological characteristics and prognosis in PDAC. The results showed that TUBA1C was significantly overexpressed in tumor tissues and associated with poor prognosis indicated by Gene Expression Profiling Interactive Analysis (GEPIA) datasets. Moreover, the TUBA1C expression was evaluated in PDAC specimens *via* immunohistochemical staining. It showed that TUBA1C was significantly highly expressed in tumor tissues compared with normal tissue. Furthermore, the clinical significance of TUBA1C was evaluated, and the differentially expressed genes (DEGs) associated with TUBA1C expression were screened from The Cancer Genome Atlas (TCGA- PDAC) datasets. Through integrative analysis, we identified that TUBA1C was associated with the cell cycle in PDAC. Additionally, functional assays were performed to verify the effects of TUBA1C knockdown on the regulation of PDAC cell malignant behaviors and its biological role in the cell cycle *in vitro*. The results demonstrated that the downregulation of TUBA1C could obviously suppress pancreatic cell proliferation and induce apoptosis *via* cell cycle. Finally, the xenograft tumor model showed that downregulation of TUBA1C could promote cell apoptosis and inhibit tumor growth *in vivo*. However, these findings may provide a basis for the development of more effective strategies for treating PDAC.

## Materials and Methods

### Datasets

Briefly, the mRNA expression profile of a total of 178 PDAC samples was obtained from TCGA- PDAC database (phs000178.v10.p8). TCGA is the largest database of molecular cancer supported by the National Cancer Institute of the United States (TCGA, http//gdc.cancer.gov/). GEPIA (http://gepiacancer-pku.cn/index.html) is a novel interactive web server that analyzes the RNA sequencing data on the basis of TCGA and the Genotype-Tissue Expression GTEx projects (22). The datasets (TCGA tumors vs. “TCGA+ GTEx normal”) were selected for the differential expression to analyze the expression level of TUBA1C in PDAC and normal pancreatic tissues according to the instructions of GEPIA. Then, the relations of disease-free survival (DFS) and overall survival (OS) rates with the expression of TUBA1C in PDAC were computed by using the GEPIA database.

### Differentially Expressed Gene Analysis

The expression profile data of 178 cases were sorted in terms of TUBA1C gene expression value. The pancreatic cancer samples were divided into the low expression group (*n* = 89) and high expression group (*n* = 89) according to the median value of TUBA1C expression. The DEGs among TUBA1C high/lower genes groups were screened by employing the edgeR package. False discovery rate (FDR) < 0.05 was used as the cutoff criteria. Pearson correlation test was conducted to determine the connection between TUBA1C and DEGs. Then, the key genes were selected to identify its relationship with the overexpression of TUBA1C by using a cutoff of *R* > 0.5.

### Kyoto Encyclopedia of Genes and Genomes Pathway Enrichment Analyses

Furthermore, the Kyoto Encyclopedia of Genes and Genomes (KEGG) pathway enrichment analysis of TUBA1C was performed to determine critical pathways closely related to TUBA1C upregulation *via* employing R package cluster Prolifer. *P* < 0.05 was considered statistically significant.

### Tissue Samples and Immunohistochemical Staining

Tissue microarrays with 99 PDAC tissues and 71 adjacent non-tumor tissues (HPan-Ade170Sur-01) were purchased from Shanghai Outdo Biotech Co. Ltd. (National Human Genetic Resources Sharing Service Platform, Shanghai, China). Ethical approval for this research was provided by the Medical Ethics Committee of Shanghai, the People's Republic of China. The whole clinicopathological data including patient age, gender, tumor position and size, pathological grade, tumor stage, lymph node metastasis, and follow-up data were analyzed. The diagnosis and staging of PDAC were confirmed through clinical evidence and pathological diagnosis according to the Staging Manual of the American Joint Committee on Cancer (AJCC) staging system, 8th edition. These patients had not been received any adjuvant chemotherapy prior to surgical resection. Each patient's clinicopathological data were obtained, and the complete data are shown in **Table 2**.

Immunochemistry (IHC) was performed to detect TUBA1C expression. Paired paraffin-embedded tissue sections of 5-μm thickness were deparaffinized and rehydrated, and then antigen retrieval was conducted in 10 mmol/L citric acid buffer at 100°C for 15 min. After incubation with anti-TUBA1C antibody (ab222849; 1:1,000, Abcam, Cambridge, UK) at 4°C overnight, these sections were rinsed with phosphate-buffered saline (PBS) and incubated with a secondary antibody at 37°C for 30 min. Then, the slides were rinsed with PBS, incubated with 3,3′-diaminobenzidine for 2 min, rinsed, and stained with hematoxylin. TUBA1C expression was observed and imaged by microscopy. All slides were separately examined and scored by three pathologists in a blindfolded method without knowledge of clinical patient data. For determining TUBA1C expression, the IHC scoring classification consolidated the evaluation of stain color intensity (0–3) with the percentage of positively stained cells (−4). The amount of these two grades was employed to classify the specimens into two groups: 0–2 scores were considered “low” expression, whereas those >2 were considered “high” expression. Scores ranged from 0 to 4 (0 for <5%, 1 for 5–25%, 2 for 26–50%, 3 for 51–75%, and 4 for 76–100%).

### Cell Lines and Cell Culture

Human pancreatic cancer cell lines consisting of AsPC-1, BxPC-3 CFPAC-1, L3.6pl, PANC-1, MIApaCa-2, SU.86.86, and one normal pancreatic cell line (HPNE) were purchased from the Shanghai Cell Bank, Chinese Academy of Sciences. The cells were cultured in Roswell Park Memorial Institute (RPMI) 1640 combined with 10% fetal bovine serum (FBS; Gibco, Carlsbad, CA, USA). The L3.6pl cell line was cultured in Minimum Essential Medium (MEM) (Gibco, Grand Island, NY, USA) with 10% FBS (Gibco, Carlsbad, CA, USA). All cells were maintained at 37°C in 5% CO_2_ incubator and passaged adopting standard cell culture techniques.

### Small Interfering RNA Transfection

The sequences of the small interfering RNA (siRNA) specifically targeting TUBA1C and its negative control (NC) were synthesized by GenePharma (Shanghai, China). The sequences of siRNA1 (TUBA1C-si1), siRNA2 (TUBA1C-si2), and siRNA3 (TUBA1C-si3) were as follows: siRNA15′-GAACCUGGCUGUGAUUCAATT-3′; siRNA25′-CGAACAGCUUACUGUAGCATT-3′; and siRNA35′-GGAAUUAGAUCCUUCAAAUTT-3′. A negative siRNA was used as the control (NC, 5′-UUCUCCGAACGUGUCACGUTT-3′). The AsPC-1 and BxPC-3 cells were transfected at a concentration of 10 nM using Lipofectamine 2000 (Invitrogen; Thermo Fisher Scientific, Waltham, MA, USA) according to the manufacturer's protocol. Cells were collected 48 h after transfection. The transfected cells were used for protein extraction and other subsequent experiments as described below.

Three TUBA1C specific short hairpin (sh) RNA lentiviruses (sh1, sh2, and sh3) and vector were purchased from Genomeditech Biotechnology (Shanghai) and presented with the following respective sequences: CCCAACCTACACTAACCTTAA, GCAAGGAAGATGCTGCCAATA, and GCTGCCCTTGAGAAGGATTAT. The sh1 sequence, which produces the most efficient knockdown of TUBA1C on BxPC-3 cell lines, was selected for the following studies. PDAC cell lines were treated with lentivirus or vector with 8 μg/ml of polybrene (Sigma, USA).

### Western Blotting

Cells were washed with PBS for fluorescent times, and total protein of the different groups was extracted using RIPA Lysis buffer (Beyotime, Shanghai, China) combined with 1% cocktail protease inhibitors (Hoffmann-La Roche Ltd., Basel, Switzerland). Then, the supernatants were collected after centrifugation (4°C, 15,000 × *g*, 15 min). Protein concentration was measured by bicinchoninic acid (BCA) protein assay (Thermo Fisher Scientific, Waltham, MA, USA). Equal amounts of protein were loaded in 12% Tris-acetate gels (Invitrogen, Thermo Fisher Scientific) and then separated by electrophoresis on sodium dodecyl sulfate–polyacrylamide gel electrophoresis (SDS-PAGE) gels. Next, the proteins were transferred onto a polyvinylidene fluoride membrane. The membrane was blocked with 5% non-fat milk in Tris-buffered saline containing 1% Tween-20 (TBST) for 1 h at room temperature and further incubated with primary antibodies for 12 h at 4°C. The primary antibodies used in this experiment were TUBA1C at 1:1,000 (Abcam, Cambridge, UK), cyclin D1, cyclin E1 at 1:2,000 (Abcam, Cambridge, UK), and cyclin-dependent kinase (CDK) 2, CDK 4, CDK 6, and GAPDH at 1:1,000 (Abcam, Cambridge, UK). After being washed with TBST three times, the membrane was incubated with secondary antibodies for 1 h at room temperature. The secondary antibodies used in this experiment were as follows: goat anti-rabbit–horseradish peroxidase (HRP; 1:5,000; cat no. PDR007; Fabio Science, Hangzhou, China) and goat anti-mouse–HRP (1:5,000; cat no. PDM007; Fabio Science). Immunodetection was performed by EZ-ECL chemiluminescence detection kit (Biological Industries, Kibbutz Beit Haemek, Israel). Protein bands were analyzed using image analysis software, and GAPDH was selected as an internal control.

### Cell Proliferation Assays

#### Cell Counting Kit-8 Assay

In brief, cells were seeded into 96-well plates at 2 × 10^4^ cells/well (Corning, NY, USA) in 100 μl of culture medium for 24 h before transfection, and the medium was incubated for 48 h after transfection. Then, Cell Counting Kit-8 (CCK-8) solution (10 μl/well; Dojindo, Kumamoto, Japan) was added, and the plates were incubated for 1 h, and the optical density (OD) values were measured daily at 450 nm for a total of four consecutive days using an ELx800 absorbance microplate reader (BioTek, USA). The absorbance was recorded with an automated plate reader.

#### Colorimetric Immunoassay

Cell proliferation was assessed using a colorimetric immunoassay (Cell-Light™ EdU Apollo567 *in vitro* Imaging Kit; RiboBio, Guangzhou, People's Republic of China) according to the manufacturer's instructions. Cells were cultured in a confocal dish at a density of 1 × 10^5^ cells per dish for 24 h before transfection and incubated for 48 h after transfection. Then, immunoassay was performed.

#### Cell Apoptosis Assay

Cells were seeded into 6-well plates at 5 × 10^5^ cells/well. Forty-eight hours following transfection, the cells were harvested, washed three times with PBS, and resuspended in a staining solution containing annexin V-fluorescein isothiocyanate and propidium iodide. Cell apoptosis was measured by flow cytometry (BD Biosciences, San Jose, CA, USA) and analyzed using FlowJo (Tree Star) on the basis of the manufacturer's protocol.

#### Colony Formation Assay

For the colony formation assay, cells transfected with shRNA-1 were seeded into 6-well plates (2,000 cells/well). Cells were cultured in medium containing 10% FBS (Gibco, Carlsbad, CA, USA) for 12 days. The cells were washed with PBS before being fixed with methanol and stained with 1% crystal violet.

### Cell Cycle Analysis

Cell cycle distribution was analyzed by flow cytometry. Cells were cultured into 6-well plates and harvested after 48 h of transfection. Briefly, 1 × 10^6^ cells were fixed overnight in cold 70% ethanol at 4°C. After being washed with PBS, cells were resuspended in a cell cycle staining kit (Multiscience, Hangzhou, People's Republic of China) and incubated for 30 min at 37°C in the dark before analysis. Then cells were measured by using flow cytometry (Cytomics FC 500, Beckman Coulter, Miami, FL, USA) and analyzed using Modfit LT software (Verity Software House, Topsham, ME, USA).

### Transwell Assay

To determine the invasion ability of PDAC cells, a transwell assay was performed. Transwell chambers with 8 μM of pore size (Sigma-Aldrich Co. Ltd) were placed on a 24-well plate with 40 μl of Matrigel (1.5 mg/L; BD Biosciences, San Jose, CA, USA). The Matrigel was coated on the upper chamber of the bottom membrane of the transwell chamber after being diluted at 1:8 and placed in an incubator at 37°C for 30 min to polymerize the Matrigel into a gel. A cell at a density of 1 × 10^5^ was suspended in 200 μl of serum-free medium and added to the upper layer of a transwell chamber, and 600 μl of 10% FBS was added to the lower chamber. After incubation for 48 h at 37°C, the cells that did not migrate or invade through the transwell chamber were gently removed with a cotton swab. The chamber was dried and fixed with 10% formaldehyde solution (Beyotime, Shanghai, China) and stained with 0.5% crystal violet hydrate solution (Beyotime, Shanghai, China). Then, the cells were randomly elected from five fields and counted using a fluorescent microscope (magnification, × 200). The percentage of invasion cells was calculated relative to that of the control cells.

### Wound Healing Assay

A wound healing assay was performed to evaluate PDAC cell mobility. AsPC-1 or BxPC-3 cells were seeded in 6-well plates at 5 × 10^5^ cells/well. The medium was discarded after 24 h, and a straight gap was created using a sterile plastic 200-μl micropipette tip in the middle of the well. The plates were washed twice with PBS to remove floating cells and incubated at 37°C with 5% CO_2_ in a humidified atmosphere. Wound closure was observed after incubation for 24 and 48 h; thereafter, wound closure was imaged and analyzed using a light microscope (Olympus, Tokyo, Japan) and Photoshop 7.0 software (Adobe, San Jose, USA). The narrow distances between the edges of cells migrating from both sides were measured relative to that of the control cells.

### Tumor Xenograft Experiments

For xenografts, male nude mice (BALB/C) (4–5 weeks old) were obtained from (Shanghai SLAC Laboratory Animal Co., Ltd, China). All experimental procedures were approved by the Zhejiang Medical Experimental Animal Care Commission. The mice were randomly divided into two groups (6 mice/group) and were maintained under a specific pathogen-free facility according to the guidelines of laboratory animal care. Briefly, BxPC-3 cells were infected with sh-control lentivirus and sh-TUBA1C lentivirus to construct stable cell lines. BxPC-3 cells with stable expression of control shRNA or TUBA1C shRNAs were applied. These stable cells were collected and suspended in PBS. A total volume of 0.2 ml of PBS containing 1 × 10^6^ BxPC-3 cells was injected subcutaneously into the right flank of mice (*N* = 6). The mice were examined once every 3 days. Then mice were euthanized 30 days later, and the relevant data (weight, length, and width of the tumor) were reported. The tumor volume was measured employing the following formula: volume = (*L* × *W*^2^)/2, where *W* and *L* were the shortest and longest diameters of the tumor, respectively. Following euthanasia of nude mice, the tumors were surgically excised, measured, weighed, and paraffin embedded.

For immunohistochemistry, tumors from mice were paraffin embedded and immunostained. The following 6.0-μm sections were split and dominated to IHC analyzed using an anti-Ki-67 (1:125, Abcam, Cambridge, UK). The proliferation index was quantized by scoring the proportion of Ki-67-positive cells. Moreover, TUNEL Apoptosis Assay Kit (Beyotime, China) was employed to detect the apoptosis of xenograft tumors, and the apoptotic index was measured by the percentage of TUNEL-positive cells.

### Statistical Analyses

All data were presented as the mean ± standard derivation (SD). SPSS 19.0 software (SPSS, Chicago, IL, USA) was applied for the statistical analyses. The Mann–Whitney *U* test was applied to compare the expression of TUBA1C between PDAC tissues and paired adjacent non-tumor tissues. The expression of TUBA1C and clinicopathological data were analyzed using the χ^2^ test. The OS rate was evaluated by applying the Kaplan–Meier curves method, the log-rank test was used for assessing the differences in survival, and the Cox regression model was employed to investigate the prognostic agents. Differences between groups were determined using Student's *t*-test. *P* < 0.05 was considered statistically significant.

## Results

### TUBA1C Overexpression Is Correlated With Pancreatic Ductal Adenocarcinoma Progression and Poor Prognosis

**Figure 1:**
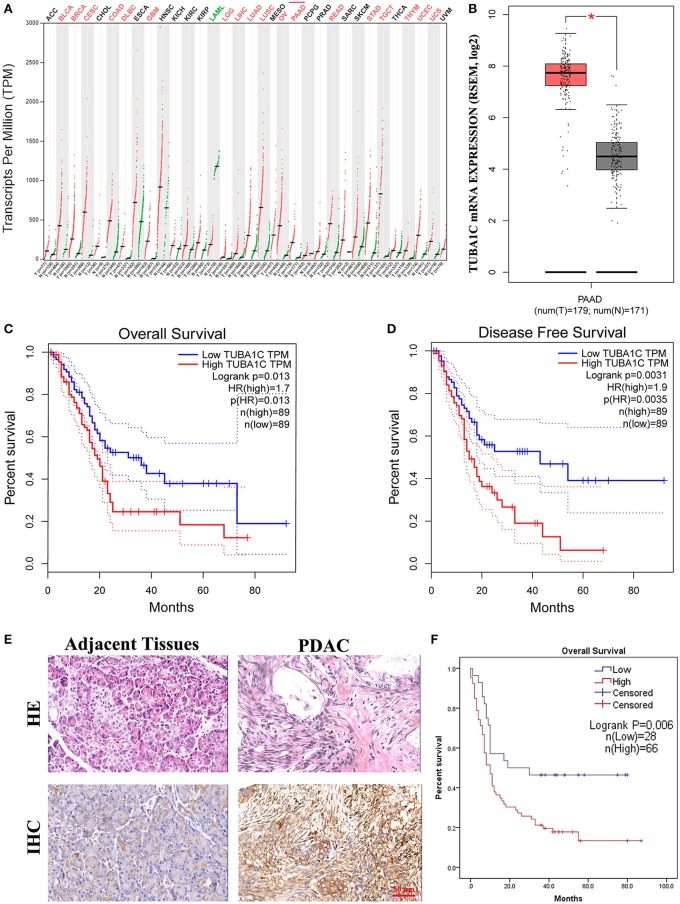
The expression of TUBA1C in pancreatic ductal adenocarcinoma (PDAC) tissues. **(A)** TUBA1C mRNA expression levels in different tumor tissues obtained from the Gene Expression Profiling Interactive Analysis (GEPIA) database. The red dot and green dot represent the tumor sample and normal sample, respectively. A red label indicates that TUBA1C is upregulated in this cancer compared with its normal tissues, whereas the green denotes downregulation. A black label denotes that no significant difference was observed between tumor and normal tissue in this disease. **(B)** The expression level of TUBA1C was upregulated in PDAC (*n* = 179) compared with normal tissues (*n* = 171). **(C,D)** The overall survival and disease-free survival in high and low TUBA1C group were computed by GEPIA; high expression of TUBA1C correlated with poor prognosis of PDAC patients (*n* = 178). Dashed line represents 95% confidence interval. **(E)** Representative HE and immunohistochemistry staining for PDAC tissues and adjacent pancreatic tissues. **(F)** Kaplan–Meier survival curves according to the expression level of TUBA1C in PDAC indicated that the high expression level of TUBA1C was significantly correlated with shorter overall survival (*n* = 94; *P* = 0.006). Five cases could not be contacted, excluding survival analysis.

To investigate the role of TUBA1C in patients with PDAC, bioinformatic analysis was employed. The results showed that the mRNA expression level of TUBA1C was upregulated in various malignancies (including PDAC), which is shown by the red label in Figure 1A, whereas low expression of TUBA1C was only observed in acute myeloid leukemia (TCGA-LAML) by the GEPIA database (Figure 1A). Similarly, the level of TUBA1C was obviously elevated in PDAC tissues compared with paired TCGA- PDAC samples and GTEx normal pancreatic samples (*P* < 0.05, Figure 1B). Kaplan–Meier survival analysis of data from TCGA showed that pancreatic cancer patients with higher expression of TUBA1C had shorter OS and DFS (Figures 1C,D). Furthermore, the expression of TUBA1C was determined using tissue microarray-based immunohistochemistry with 99 human pancreatic tumor samples and 71 adjacent tissues. Positive staining was observed in the cytoplasm of the pancreatic tumor cells, and the level of TUBA1C staining was significantly upregulated in PDAC tissue compared with adjacent non-tumor tissues (Figure 1E, Table 1). And the correlation between the TUBA1C expression and clinicopathological variables was analyzed. The statistical result indicated that TUBA1C expression was significantly associated with the p53 (*P* = 0.008) (Table 2). Besides, the Kaplan–Meier method analysis and log-rank test revealed that the higher expression of TUBA1C was significantly correlated with shorter OS (*P* = 0.006) (Figure 1F). Our findings were consistent with the analysis of TCGA data. Moreover, univariate analysis demonstrated close connection between OS rate and tumor grade [hazard ratio (HR) = 1.954; 95% confidence interval (95% CI) = 1.201–3.178; *P* = 0.007), N stage (HR = 1.976; 95% CI = 1.208–3.233; *P* = 0.007), TNM stage (HR = 1.567; 95% CI = 1.111–2.210; *P* = 0.011), and expression of TUBA1C (HR = 2.147; 95% CI = 1.211–3.809; *P* = 0.009), whereas multivariate analysis showed that the relation between tumor grade (HR = 2.277; 95% CI = 1.332–3.893; *P* = 0.003), N stage (HR = 2.111; 95% CI = 1.204–3.701; *P* = 0.009), and TUBA1C expression (HR = 1.838; 95% CI = 1.009–3.349; *P* = 0.047) (Table 3).

**Table 1:** Differential expression of TUBA1C in pancreatic cancer and adjacent tissues.

	***n***	**TUBA1C expression**		**Chi-square value**	***p-*value**
		**High (%)**	**Low (%)**		
Pancreatic cancer	65	45	20	12.381	0.000^*^
Adjacent tissues	65	25	40		

* *Statistically significant (p < 0.05)*.

**Table 2:** Correlation between TUBA1C expression and clinicopathological characteristics.

	**Variables**	**TUBA1C expression**		**Total**	**χ^2^**	***p-*value**
		**High**	**Low**			
Age (year)					0.026	0.871
	≤60	33	14	47		
	>60	33	13	46		
	dull			1		
Sex					1.818	0.178
	Female	21	13	34		
	male	45	15	60		
Grade					0.774	0.379
	I/II	46	22	68		
	III	20	6	26		
T stage					0.163	0.686
	T1/T2	23	51	74		
	T3	5	14	19		
	dull			1		
N stage					0.118	0.731
	N0	37	14	51		
	N1	27	12	39		
	dull			4		
M stage					0.022	0.881
	M0	64	28	92		
	M1	2	0	2		
TNM stage					0.029	0.866
	I/II	62	28	90		
	IV	2	0	2		
	Dull			2		
Smoke					0.749	0.387
	no	8	1	9		
	yes	2	0	2		
	dull			83		
Alcohol					0.749	0.387
	no	8	1	9		
	yes	2	0	2		
	dull			83		
Diabetes					0.286	0.592
	no	7	1	8		
	yes	3	0	3		
	dull			83		
p53					6.973	0.008^*^
	negative	17	15	32		
	positive	44	11	55		
	dull			7		
Hepatitis					0.749	0.387
	no	8	1	9		
	yes	2	0	2		
	dull			83		
Ki67					1.363	0.243
	negative	15	10	25		
	positive	43	16	59		
	dull			10		

*Statistical analyses were performed by the Chi-square test*.

* *P < 0.05 was considered statistically significant*.

**Table 3:** Univariate and multivariate analyses of the factors correlated with Overall survival of Pancreatic carcinoma patients.

**Variables in the Equation**						
**Variables**	**Univariate analysis**			**Multivariate analysis**		
	**HR**	**95%CI**	***p-*****value**	**HR**	**95%CI**	***p-*****value**
TUBA1C	2.147	1.211–3.809	0.009*	1.838	1.009–3.349	0.047*
Sex	1.153	0.703–1.891	0.572			
Grade	1.954	1.201–3.178	0.007*	2.277	1.332–3.893	0.003*
Age	1.315	0.822–2.104	0.253			
T stage	0.807	0.461–1.411	0.452			
N stage	1.976	1.208–3.233	0.007*	2.111	1.204–3.701	0.009*
M stage	1.720	0.420–7.050	0.451			
TNM stage	1.567	1.111–2.210	0.011*	1.175	0.781–1.768	0.440
smoke	0.687	0.082–5.742	0.729			
alcohol	0.687	0.082–5.742	0.729			
diabetes	1.018	0.196–5.288	0.983			
p53	1.462	0.863–2.477	0.158			
Hepatitis	0.774	0.093–6.469	0.813			
Ki67	0.868	0.505–1.492	0.609			

*Univariate and multivariate analyses of the factors correlated with Overall survival of PDAC patients*.

* *P Statistically significant (P <0.05) PDAC, pancreatic ductal adenocarcinoma; HR, hazard ratio; CI, confidence interval; TNM, tumor-node-metastasis*.

### Differential Expression and Kyoto Encyclopedia of Genes and Genomes Pathway Enrichment Analysis

**Figure 2:**
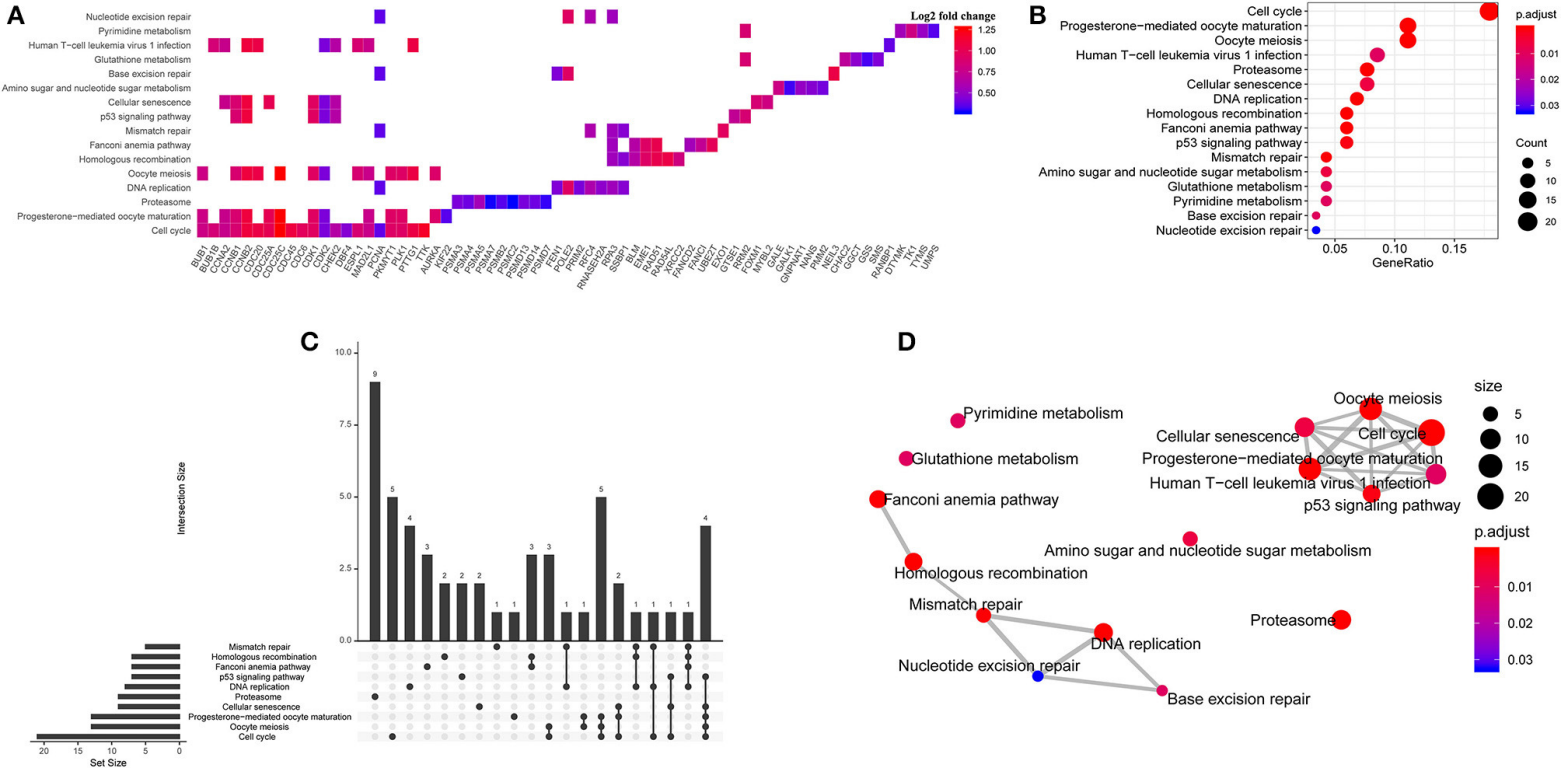
Bioinformatic analysis for potential signaling pathways. **(A)** The enriched pathways of upregulated differentially expressed genes (DEGs). **(B)** The Kyoto Encyclopedia of Genes and Genomes (KEGG) enrichment analysis in the TCGA- PDAC datasets. **(C)** Upset diagram of intersection of DEGs among enriched pathways. **(D)** The correlation network of KEGG pathways.

To further explore the potential relation between gene expression profile/KEGG pathway and TUBA1C expression, 178 samples of TCGA- PDAC dataset were divided into high group and low group according to the TUBA1C mRNA expression level. Then, the edgeR package was applied to identify the DEGs in the two groups with the cutoff value of FDR < 0.05; 3,493 upregulated genes and 2,731 downregulated genes were identified (Supplementary Table 1). Furthermore, Pearson tests with the cutoff value of *R* > 0.5 and *P* < 0.05 were conducted, and the data indicated that a total of 233 DEGs were positively correlated with TUBA1C expression. Then, KEGG enrichment pathway analysis was implemented. The result showed that these DEGs were significantly enriched in a series of cellular pathways including cell cycle, progesterone-mediated oocyte maturation, oocyte meiosis, proteasome, DNA replication, p53, and other pathways; and the cell cycle was the most significant pathway (Figures 2A–D).

### The Association Between TUBA1C Co-expression Cell Cycle Regulation Genes and the Prognosis of Pancreatic Ductal Adenocarcinoma Patients

We determined the impact of cell cycle pathway co-expression genes on PDAC patients' prognosis. Consequently, 21 genes that were enriched in the pathway were observed by the Oncolnc database (www.oncolnc.org). Then univariate Cox regression and log-rank test were employed, and the FDR correction was calculated. Eight genes (BUB1, BUB1B, CCNA2, CCNB2, CDC6, CDC20, CDK1, and TTK) among 21 common network genes were correlated with worse survival of pancreatic cancer, highlighted with *P*-value significantly <0.05 (Figure 2A, Supplementary Table 2, Supplementary Figure 1).

### Expression of TUBA1C in Pancreatic Cancer Cell Lines

**Figure 3:**
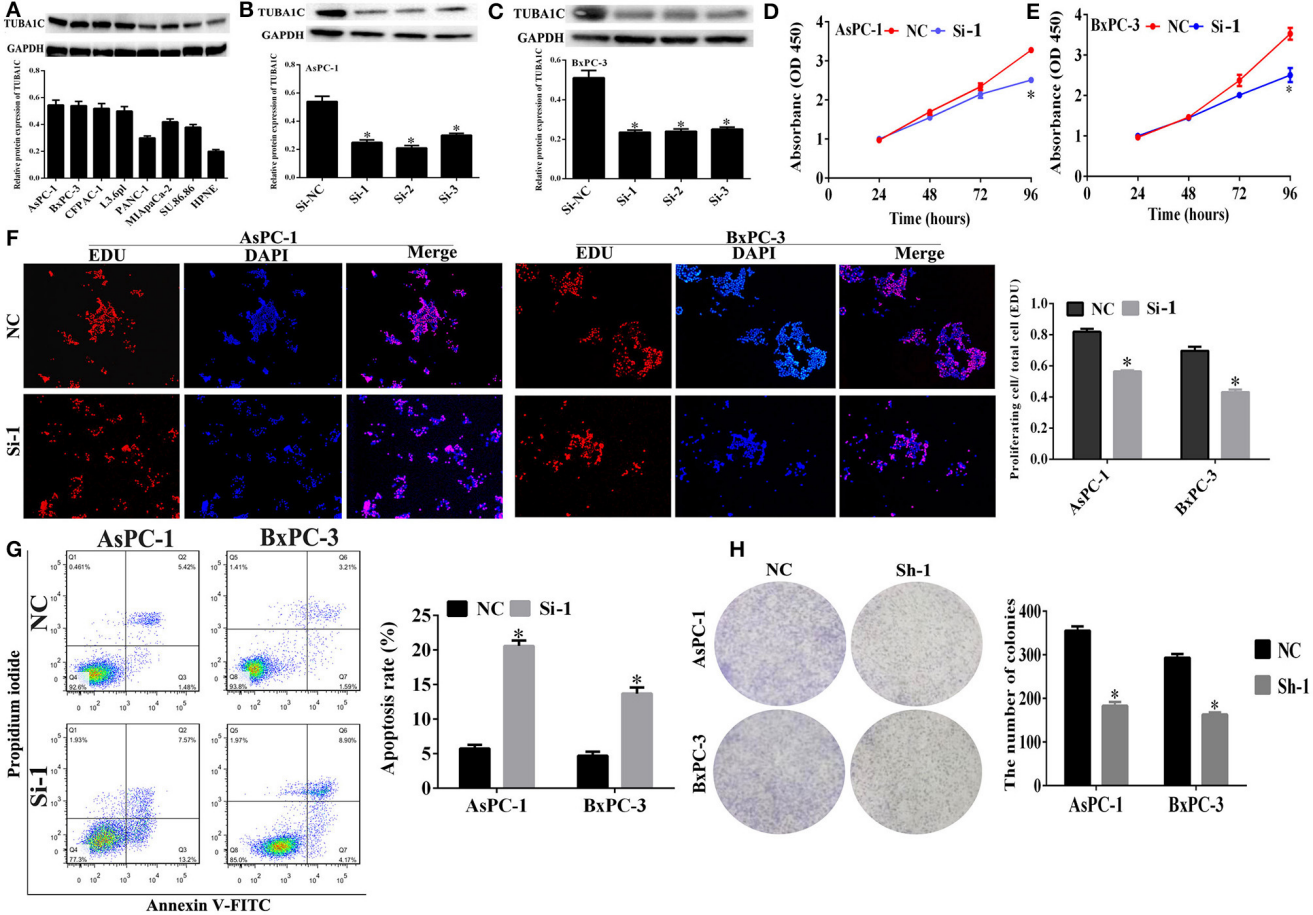
The effect of TUBA1C knockdown on cell viability and apoptosis in pancreatic ductal adenocarcinoma (PDAC) cells *in vitro*. **(A)** TUBA1C protein expression levels in normal pancreatic cell line (HPNE) and seven PDAC cell lines. **(B,C)** Evaluation of TUBA1C expression in AsPC-1 and BxPC-1 cells after siRNA transfection. **(D,E)** The effect of TUBA1C knockdown on proliferation ability of AsPC-1 and BxPC-3 cell lines detected by Cell Counting Kit-8 (CCK-8) assay and **(F)** EdU assay. **(G)** The effect of TUBA1C knockdown on cell apoptosis in AsPC-1 and BxPC-3 cells determined by flow cytometry. **(H)** The colony formation assay for evaluation of the proliferation of AsPC-1 and BxPC-3 cells after shRNA transfection. All results are displayed as the mean ± SD (*n* = 3; *P < 0.05) compared with the negative control (NC) group.

Western blotting (WB) assay was employed to evaluate the expression of TUBA1C in pancreatic cancer cell lines. The results showed that TUBA1C was significantly overexpressed in pancreatic cancer cell lines (including AsPC-1, BxPC-3, CFPAC-1, L3.6pl, PANC-1, MIApaCa-2, and SU.86.86) than normal pancreatic cell line (HPNE) as shown in Figure 3A. Because the AsPC-1 and BxPC-3 cell lines had high protein levels of expression of TUBA1C, those two cell lines were selected for the subsequent *in vitro* experiments. TUBA1C was knockdown in AsPC-1 and BxPC-3 cells by transfected with TUBA1C-siRNA#1–3 or control siRNAs. WB assay verified that transfection with siRNAs#1, #2, or #3 effectively suppressed TUBA1C expression compared with transfected control NC siRNAs (*P* < 0.05, Figures 3B,C). SiRNA-1 targeting TUBA1C was used for the subsequent experiments to investigate the role of TUBA1C in the development and progression of PDAC.

### Effects of TUBA1C Knockdown on Cell Proliferation and Apoptosis in Pancreatic Ductal Adenocarcinoma Cells *in vitro*

The CCK-8 assay was employed to detect the effect of TUBA1Con PDAC cell proliferation at indicated times (0, 24, 48, and 72 h). The results showed that the “AsPC-1 and BxPC-3” cell proliferation was significantly decreased compared with the NC (*P* < 0.05, Figures 3D,E). In addition, CCK-8 assay revealed that downregulation or overexpression of TUBA1C had no effect on the proliferation of HPNE cells. Moreover, the overexpression of TUBA1C had no significant impact on the migration and invasion of HPNE cells. Additional functional examination for HPNE cell lines was not pursued (Supplementary Figure 2). Meanwhile, the EdU assay and flow cytometry were employed to determine the effect of TUBA1C downregulation on cell proliferation and apoptosis changes in PDAC cells, respectively. The results confirmed that knockdown of TUBA1C significantly inhibited the pancreatic cancer cell proliferation (AsPC-1 and BxPC-3) (*P* < 0.05, Figure 3F) and enhanced the cell apoptosis in AsPC-1 and BxPC-3 cell lines (*P* < 0.05, Figure 3G). In addition, the colony formation assay was conducted to further verify that TUBA1C downregulation has an effect on PDAC cell proliferation. The results indicated that the downregulation of TUBA1C significantly inhibited colony formation in both AsPC-1 and BxPC-3 cell lines (*P* < 0.05, Figure 3H).

### Effects of TUBA1C Knockdown on Cell Cycle in Pancreatic Ductal Adenocarcinoma Cells

**Figure 4:**
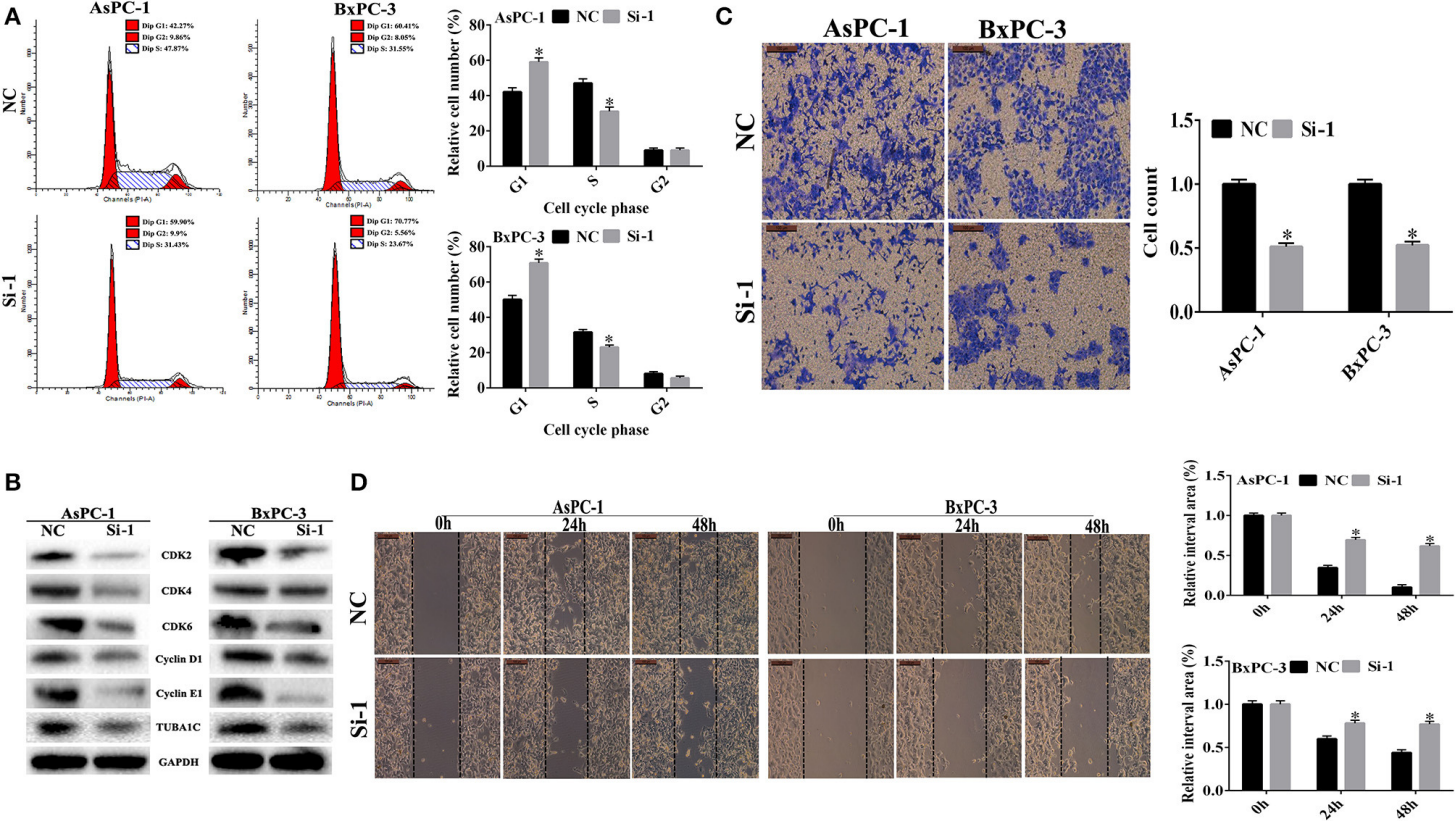
The effect of TUBA1C knockdown on cell cycle, invasion, and migration in pancreatic ductal adenocarcinoma (PDAC) cells *in vitro*. **(A)** Cell cycle analysis using flow cytometry in AsPC-1 and BxPC-3 cells following TUBA1C knockdown. **(B)** Western blotting assay for cell cycle relative proteins. **(C,D)** Representative images of transwell assays and wound healing assay, and the quantitative result after TUBA1C knockdown in AsPC-1 and BxPC-3 cells. All results are displayed as the mean ± SD (*n* = 3; *P < 0.05) compared with the negative control (NC) group.

Cell cycle analysis was performed to evaluate the effect of TUBA1C expression on the cell cycle using flow cytometry. As shown in Figure 4A, the downregulation of TUBA1C in both AsPC-1 and BxPC-3 cells significantly increased the percentage of cells in the G1 phase and decreased the rate of cells in the S phase (*P* < 0.05). These data suggested that TUBA1C downregulation induced PDAC cells arrest at the G0/G1 phase and may block cell cycle transition from G1 to S phase. Additionally, protein expression levels of cell cycle regulators were measured by WB analysis. The results demonstrated that the cell cycle proteins including CDK2, CDK4, CDK6, cyclin D1, and cyclin E1 were simultaneously suppressed in the TUBA1C knockdown groups (*P* < 0.05, Figure 4B).

### Knockdown of TUBA1C Inhibited Pancreatic Ductal Adenocarcinoma Cell Invasion and Migration *in vitro*

In order to investigate the effects of the knockdown of TUBA1C on PDAC cell migration and invasion, a transwell assay using Matrigel was performed following the transfection of siRNA TUBA1C or control siRNA NC into AsPC-1 and BxPC-3 cell lines. The results showed that invasion was significantly decreased after knockdown of TUBA1C relative to that of the control NC group for wound healing assay (*P* < 0.05, Figure 4C). The results showed that TUBA1C knockdown had significantly reduced the migration ability of PDAC cells after scratching at 24 and 48 h, compared with the control NC group (*P* < 0.05, Figure 4D). These results indicated that the expression of TUBA1C could influence the migration and invasion of PDAC cells.

### Knockdown of TUBA1C Suppressed Tumorigenicity *in vivo*

**Figure 5:**
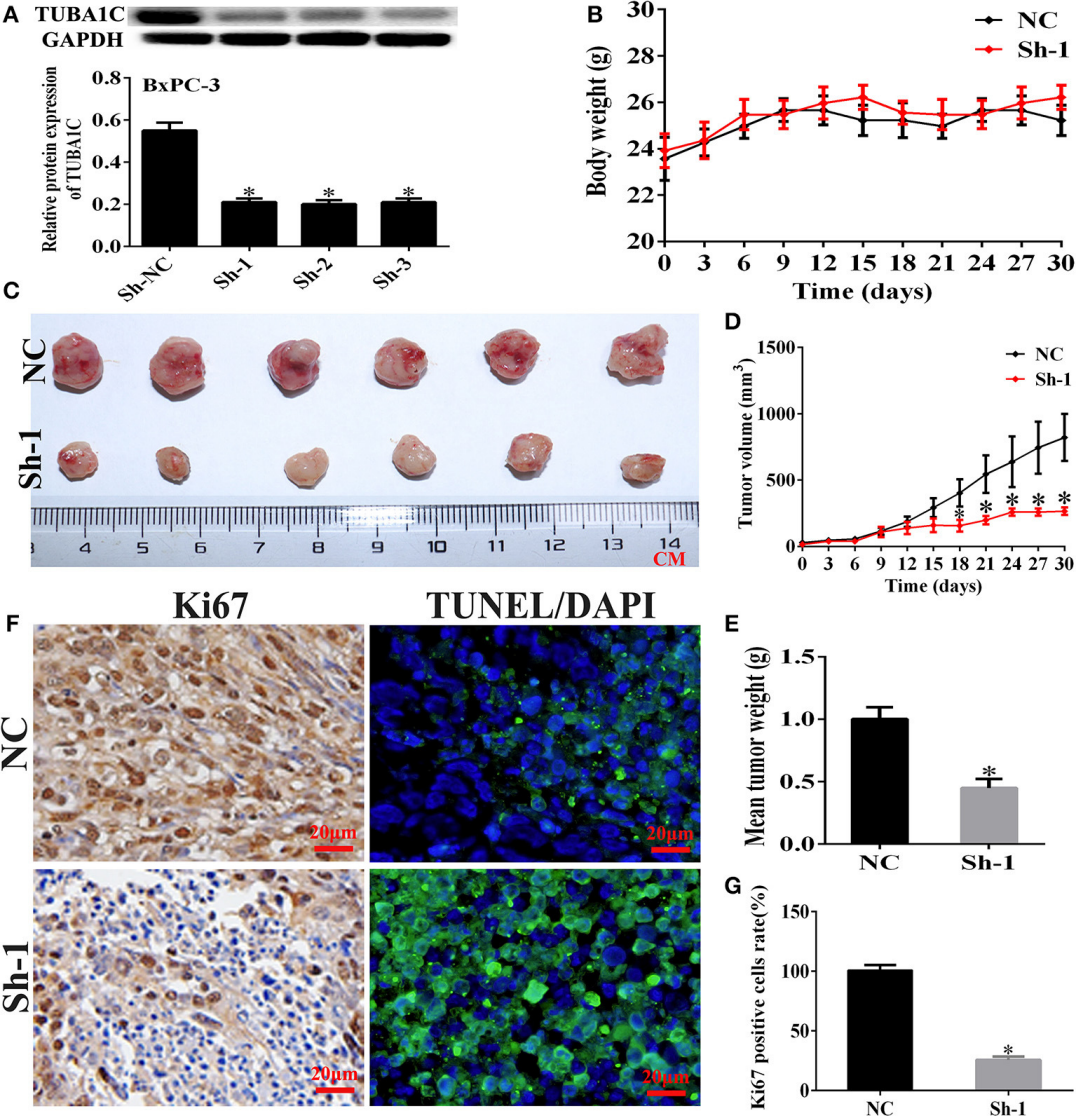
Knockdown of TUBA1C suppressed tumorigenicity *in vivo*. **(A)** Evaluation of TUBA1C knockdown in BxPC-3 cell lines after transfection with TUBA1C shRNA by western blotting. **(B)** Body weight of mice in each group. **(C–E)** Representative effect of TUBA1C knockdown on tumor growth (tumor volume and weight) (*n* = 6). **(F)** Representative staining images for Ki-67 stained (brown) and TUNEL positive cells (green) counterstained with DAPI (blue) in different groups. **(G)** Percentage of Ki-67-positive cells in different groups (magnification × 400). ImageJ software was used to calculate the Ki-67-positive cells (*n* = 3). The results from the xenograft tumor model are presented as the mean ± SD (*P < 0.05) compared with the negative control (NC) group.

To investigate the biological effects of TUBA1C on PDAC progression *in vivo*, with the use of WB, the BxPC-3 cells were transfected with the use of WB, the BxPC-3 cells were transfected with non-targeting shRNA (NC) or TUBA1C sh1, sh2, and sh3 (Figure 5A). The BxPC-3 cell line xenograft model was applied to verify the tumorigenicity of TUBA1C *in vivo*. Control and sh1 were injected subcutaneously into male nude mice. The sh-TUBA1C group revealed significant inhibition of tumor growth compared with that of the control group (Figures 5B–E). Furthermore, IHC analysis revealed that the knockdown of TUBA1C showed a lower Ki-67 proliferation index and an increased percentage of TUNEL-positive apoptotic cells than did control (Figures 5F,G). Collectively, our findings showed that the knockdown of TUBA1C suppresses tumor growth *in vivo*.

## Discussion

PDAC is a highly malignant disease with aggressive growth and early metastatic dissemination, and ~250,000 people die annually worldwide owing to the absence of clinically informative and diagnostic biomarkers (4, 23). The accumulation of clinically related information for detecting PDAC in the early/premalignant stage will conduce to reduce the mortality of PDAC. Hence, it is crucial to develop prognostic markers that assist clinicians in electing therapeutic targets for treating PDAC. TUBA1C is a subtype of α-tubulin correlated with the organization of microtubule structure, which is a multifunctional cytoskeletal protein involved in critical cellular roles and essential in the process of mitosis and cell division (24, 25). Some researches indicated that TUBA1C was implicated in cell proliferation and cell cycle in numerous cancers (26, 27). Meanwhile, previous studies demonstrated TUBA1C upregulation could significantly impact tumor growth and progression (28, 29). In addition, it was reported that TUBA1C has emerged as a critical intermediary in the cell cycle signaling pathway (21). Furthermore, recently studies found that knockdown of β-tubulin enhances the ability of vinca alkaloids to arrest mitosis and consequently induce apoptosis (30). However, there were no reports about the relation between TUBA1C expression and PDAC. Accordingly, we inferred that TUBA1C might correlate to the prognosis in patients with PDAC. Therefore, the current study was focused on investigating the impact of TUBA1C overexpression on the proliferation, cell cycle distribution, potential biological functions, and prognostic significance in PDAC.

In this study, the data obtained from the TCGA and the GTEx projects through the online GEPIA database indicated that the expression of TUBA1C was upregulated and significantly correlated with DFS and OS rates in PDAC. Consistently, IHC analysis confirmed that the percentage of intense positive staining for TUBA1C expression was significantly upregulated in PDAC than in normal adjacent tissues, and the positive staining of TUBA1C was particularly in the cytoplasm. Furthermore, it was found that the expression of TUBA1C was correlated with p53 expression. Previous studies reported that complex changes in tubulin isotype expression and microtubule dynamics were associated with p53 alterations (31). Furthermore, Kaplan–Meier analysis indicated that OS was significantly shorter in PDAC patients with high expression of TUBA1C than in those with low expression of TUBA1C. In addition, the univariate analysis demonstrated that the overexpression of TUBA1C was a risk factor for the OS of PDAC patients. Meanwhile, multivariable Cox regression analysis further identified that the overexpression of TUBA1C was a significant independent predictor for OS and is associated with pathological grade, N stage, and TNM stage in patients with PDAC. Moreover, through the collection of co-expression genes and KEGG pathway analysis using the TCGA database, the preliminary results demonstrated that “cell cycle process” pathway was enriched in high expression of TUBA1C. Besides, DEG analysis also showed that 21 protein-coding genes were potentially co-expressed with TUBA1C. All data revealed that these genes were substantially enriched in the promotion of the cell cycle and associated with poor prognosis in PDAC patients. These results strongly suggested that genes co-expressed with TUBA1C may play a key role in promoting the progression of PDAC.

Further studies verified the potential role of TUBA1C in PDAC by interfering with the TUBA1C expression. The results showed that TUBA1C downregulation significantly suppressed the cell proliferation and cell cycle and markedly suppressed tumor growth and increased the apoptosis rate *in vitro* and *in vivo*. Importantly, TUBA1C downregulation had no effect on the proliferation of non-tumorigenic HPNE cell lines *in vitro*. In addition, TUBA1C knockdown dramatically decreased the ability of migration and invasion of PDAC cells *in vitro*. These results were consistent with the previous studies that TUBA1C overexpression promoted HCC cell growth and influenced the cell cycle. Furthermore, cell cycle flow cytometry assay and WB analysis were conducted to determine whether knockdown of TUBA1C decreased the ability of proliferation, migration, and invasion of PDAC cells. The results indicated that the percentage of PDAC cells in the G1 phase was significantly increased whereas the S phase was markedly decreased after TUBA1C knockdown. These findings confirmed that downregulation of TUBA1C induced cell cycle arrest at the G0/G1 phase. And the data were in line with WB analysis. Proteins and protein kinases that regulate the cell cycle (cyclins D1 and E1 and CDKs 2, 4, and 6) expression were decreased following TUBA1C knockdown in PDAC cell lines. In particular, CDK 2 may stimulate the G1/S transition when binding to cyclin E (32). CDK4 and CDK6 are necessary proteins that regulate the G1 checkpoint in cell proliferation (33). Cyclin D1 combines with CDK4/6 and stimulates CDK4/6, which consequently phosphorylates tumor suppressor retinoblastoma family members protein and allows the cell cycle to progress *via* G1 into S phase (34, 35). Furthermore, the KEGG pathway analysis and cell cycle examination suggested that TUBA1C, a component of microtubulin, might affect the proliferation and migration of PDAC cells by disturbing mitotic spindle assembly and interfering with the microtubule network. Collectively, this experiment revealed that TUBA1C downregulation might suppress the migration and invasion and might induce the cell apoptosis by regulating cell cycle signaling pathway in PDAC cells. Our findings suggested that TUBA1C may serve as a potential prognostic marker and may produce a novel molecular target for PDAC therapy.

## Conclusion

The current study revealed that the level of TUBA1C was upregulated in PDAC tissues and was correlated with the progression of PDAC and poor prognosis. TUBA1C played an essential role in PDAC cell growth, migration, and invasion, *via* regulated cell cycle signaling pathway. Our findings suggest that TUBA1C serves as a potential prognostic marker and produces a novel molecular target in cancer therapy.

## Data Availability Statement

Publicly available data was accessed from the Cancer Genome Atlas (TCGA) database (https://cancergenome.nih.gov/) and GEPIA (http://gepia.cancer-pku.cn).

## Ethics Statement

The studies involving human participants were reviewed and approved by Human Research Ethics Committee of The Second Affiliated Hospital, College of Medicine, Zhejiang University (Hangzhou, China). The patients/participants provided their written informed consent to participate in this study. The animal study was reviewed and approved by Zhejiang Medical Experimental Animal Care Commission.

## Consent For Publication

Knowledgeable permission was acquired from all individual participants included in the research.

## Author Contributions

MA designed and conceived the experiments and conducted the experiments and investigated the data. PZ and QZ produced the figures and directed and corrected the study. GL performed the detailed bioinformatic analysis. WW proposed and designed the research.

### Conflict of Interest

The authors declare that the research was conducted in the absence of any commercial or financial relationships that could be construed as a potential conflict of interest.

## Supplementary Material

Supplementary Table 1 Co-expressed genes of TUBA1C.

Supplementary Table 2 FDR correction of cell cycle-related genes in patients with PDAC.

## Abbreviations

CI: confidence interval
DEGs: differentially expressed genes
DFS: disease-free survival
DMEM: Dulbecco's modified Eagle's medium
FBS: fetal bovine serum
GEPIA: Gene Expression Profiling Interactive Analysis
HR: hazard ratio
KEGG: Kyoto Encyclopedia of Genes and Genomes
OS: overall survival
PDAC: pancreatic ductal adenocarcinoma
TCGA: The Cancer Genome Atlas
TUBA1C: α-tubulin.

## References

[1] Ryan DP Hong TS Bardeesy N Pancreatic adenocarcinoma. N Engl J Med. (2014) 371:1039–49. 10.1056/NEJMra1404198 25207767

[2] Sun H Ma H Hong G Sun H Wang J. Survival improvement in patients with pancreatic cancer by decade: a period analysis of the SEER database, 1981-2010. Sci Rep. (2014) 4:6747. 10.1038/srep06747 25339498 PMC5381379

[3] Siegel RL Miller KD Cancer statistics, 2019. (2019) 69:7–34. 10.3322/caac.21551 30620402

[4] Siegel RL Miller KD Jemal A Cancer statistics, 2016. CA Cancer J Clin. (2016) 66:7–30. 10.3322/caac.21332 26742998

[5] Waterhouse MA Burmeister EA O'Connell DL Ballard EL Jordan SJ Merrett ND. Determinants of Outcomes Following Resection for Pancreatic Cancer-a Population-Based Study. J Gastrointest Surg. (2016) 20:1471–81. 10.1007/s11605-016-3157-4 27184672

[6] Rhim AD Mirek ET Aiello NM Maitra A Bailey JM McAllister F. EMT and dissemination precede pancreatic tumor formation. Cell. (2012) 148:349–61. 10.1016/j.cell.2011.11.025 22265420 PMC3266542

[7] Teague A Lim KH Wang-Gillam A. Advanced pancreatic adenocarcinoma: a review of current treatment strategies and developing therapies. Ther Adv Med Oncol. (2015) 7:68–84. 10.1177/1758834014564775 25755680 PMC4346211

[8] Hammel P Huguet F van Laethem JL Goldstein D Glimelius B Artru P. Effect of chemoradiotherapy vs chemotherapy on survival in patients with locally advanced pancreatic cancer controlled after 4 months of gemcitabine with or without erlotinib: the LAP07 randomized clinical trial. Jama. (2016) 315:1844–53. 10.1001/jama.2016.4324 27139057

[9] Kim ND Park ES Kim YH Moon SK Lee SS Ahn SK. Structure-based virtual screening of novel tubulin inhibitors and their characterization as anti-mitotic agents. Bioorg Med Chem. (2010) 18:7092–100. 10.1016/j.bmc.2010.07.072 20810285

[10] Luduena RF. Multiple forms of tubulin: different gene products and covalent modifications. Int Rev Cytol. (1998) 178:207–75. 9348671 10.1016/s0074-7696(08)62138-5

[11] Verdier-Pinard P Wang F Burd B Angeletti RH Horwitz SB Orr GA. Direct analysis of tubulin expression in cancer cell lines by electrospray ionization mass spectrometry. Biochemistry. (2003) 42:12019–27. 10.1021/bi0350147 14556633

[12] Glotzer M. The 3Ms of central spindle assembly: microtubules, motors and MAPs. Nat Rev Mol Cell Biol. (2009) 10:9–20. 10.1038/nrm2609 19197328 PMC2789570

[13] Stearns ME Brown DL. Microtubule organizing centers (MTOCs) of the alga Polytomella exert spatial control over microtubule initiation *in vivo* and *in vitro*. J Ultrastruct Res. (1981) 77:366–78. 7198693 10.1016/s0022-5320(81)80033-0

[14] Hetland TE Hellesylt E Florenes VA Trope C Davidson B Kaern J. Class III beta-tubulin expression in advanced-stage serous ovarian carcinoma effusions is associated with poor survival and primary chemoresistance. Hum Pathol. (2011) 42:1019–26. 10.1016/j.humpath.2010.10.025 21315408

[15] Koh Y Jang B Han SW Kim TM Oh DY Lee SH. Expression of class III beta-tubulin correlates with unfavorable survival outcome in patients with resected non-small cell lung cancer. J Thorac Oncol. (2010) 5:320–5. 10.1097/JTO.0b013e3181ce684f 20087230

[16] Lobert S Jefferson B Morris K. Regulation of beta-tubulin isotypes by micro-RNA 100 in MCF7 breast cancer cells. Cytoskeleton (Hoboken). (2011) 68:355–62. 10.1002/cm.20517 21634028

[17] Shimizu A Kaira K Yasuda M Asao T Ishikawa O. Decreased expression of class III beta-tubulin is associated with unfavourable prognosis in patients with malignant melanoma. Melanoma Res. (2016) 26:29–34. 10.1097/cmr.0000000000000208 26426765

[18] Massari F Bria E Ciccarese C Munari E Modena A Zambonin V. Prognostic value of Beta-Tubulin-3 and c-Myc in muscle invasive urothelial carcinoma of the bladder. PLoS ONE. (2015) 10:e0127908. 10.1371/journal.pone.0127908 26046361 PMC4457798

[19] Hall JL Cowan NJ. Structural features and restricted expression of a human alpha-tubulin gene. Nucleic Acids Res. (1985) 13:207–23. 10.1093/nar/13.1.207 3839072 PMC340985

[20] Lu C Zhang J He S Wan C Shan A Wang Y. Increased alpha-tubulin1b expression indicates poor prognosis and resistance to chemotherapy in hepatocellular carcinoma. Dig Dis Sci. (2013) 58:2713–20. 10.1007/s10620-013-2692-z 23625295

[21] Wang J Chen W Wei W Lou J. Oncogene TUBA1C promotes migration and proliferation in hepatocellular carcinoma and predicts a poor prognosis. Oncotarget. (2017) 8:96215–96224. 10.18632/oncotarget.21894 29221200 PMC5707094

[22] Tang Z Li C Kang B Gao G Li C Zhang Z. GEPIA: a web server for cancer and normal gene expression profiling and interactive analyses. Nucleic Acids Res. (2017) 45:W98–102. 10.1093/nar/gkx247 28407145 PMC5570223

[23] Taniuchi K Yawata T Tsuboi M Ueba T Saibara T. Efficient delivery of small interfering RNAs targeting particular mRNAs into pancreatic cancer cells inhibits invasiveness and metastasis of pancreatic tumors. Oncotarget. (2019) 10:2869–2886. 10.18632/oncotarget.26880 31080558 PMC6499602

[24] Gilbertson-White S Perkhounkova Y Saeidzadeh S Hein M Dahl R Simons-Burnett A. Understanding symptom burden in patients with advanced cancer living in rural areas. Oncol Nurs Forum. (2019) 46:428–441. 10.1188/19.onf.428-441 31225835 PMC6642634

[25] Jordan MA Wilson L. Microtubules as a target for anticancer drugs. Nat Rev Cancer. (2004) 4:253–65. 10.1038/nrc1317 15057285

[26] Bates D Eastman A. Microtubule destabilising agents: far more than just antimitotic anticancer drugs. Br J Clin Pharmacol. (2017) 83:255–268. 10.1111/bcp.13126 27620987 PMC5237681

[27] Katsetos CD Draber P. Tubulins as therapeutic targets in cancer: from bench to bedside. Curr Pharm Des. (2012) 18:2778–92. 10.2174/138161212800626193 22390762

[28] Nami B Wang Z. Genetics and expression profile of the tubulin gene superfamily in breast cancer subtypes and its relation to taxane resistance. Cancers (Basel). (2018) 10:274. 10.3390/cancers10080274 30126203 PMC6116153

[29] Li H Jiang X Zhu S Sui L. Identification of personalized dysregulated pathways in hepatocellular carcinoma. Pathol Res Pract. (2017) 213:327–332. 10.1016/j.prp.2017.01.015 28215647

[30] Sharbeen G McCarroll J Liu J Youkhana J Limbri LF Biankin AV. Delineating the role of betaIV-Tubulins in pancreatic cancer: betaIVb-tubulin inhibition sensitizes pancreatic cancer cells to vinca alkaloids. Neoplasia. (2016) 18:753–764. 10.1016/j.neo.2016.10.011 27889644 PMC5126129

[31] Galmarini CM Kamath K Vanier-Viornery A Hervieu V Peiller E Falette N. Drug resistance associated with loss of p53 involves extensive alterations in microtubule composition and dynamics. Br J Cancer. (2003) 88:1793–9. 10.1038/sj.bjc.6600960 12771997 PMC2377136

[32] Tsai LH Harlow E Meyerson M. Isolation of the human cdk2 gene that encodes the cyclin A- and adenovirus E1A-associated p33 kinase. Nature. (1991) 353:174–7. 10.1038/353174a0 1653904

[33] Ye D Luo H Lai Z Zou L Zhu L Mao J. ClC-3 chloride channel proteins regulate the cell cycle by Up-regulating cyclin D1-CDK4/6 through suppressing p21/p27 expression in nasopharyngeal carcinoma cells. Sci Rep. (2016) 6:30276. 10.1038/srep30276 27451945 PMC4959003

[34] Harbour JW Luo RX Dei Santi A Postigo AA Dean DC. Cdk phosphorylation triggers sequential intramolecular interactions that progressively block Rb functions as cells move through G1. Cell. (1999) 98:859–69. 10499802 10.1016/s0092-8674(00)81519-6

[35] Lundberg AS Weinberg RA. Functional inactivation of the retinoblastoma protein requires sequential modification by at least two distinct cyclin-cdk complexes. Mol Cell Biol. (1998) 18:753–61. 10.1128/mcb.18.2.753 9447971 PMC108786

