# Supplementary material for: Upregulated Expression of TUBA1C Predicts Poor Prognosis and Promotes Oncogenesis in Pancreatic Ductal Adenocarcinoma via Regulating the Cell Cycle
Source: Front Oncol. 2020 Feb 14;10:49. doi: 10.3389/fonc.2020.00049 (PMC7033491; doi:10.3389/fonc.2020.00049)
Supplement: Supplementary file 1 [file Data_Sheet_1.pdf]

Supplementary Material

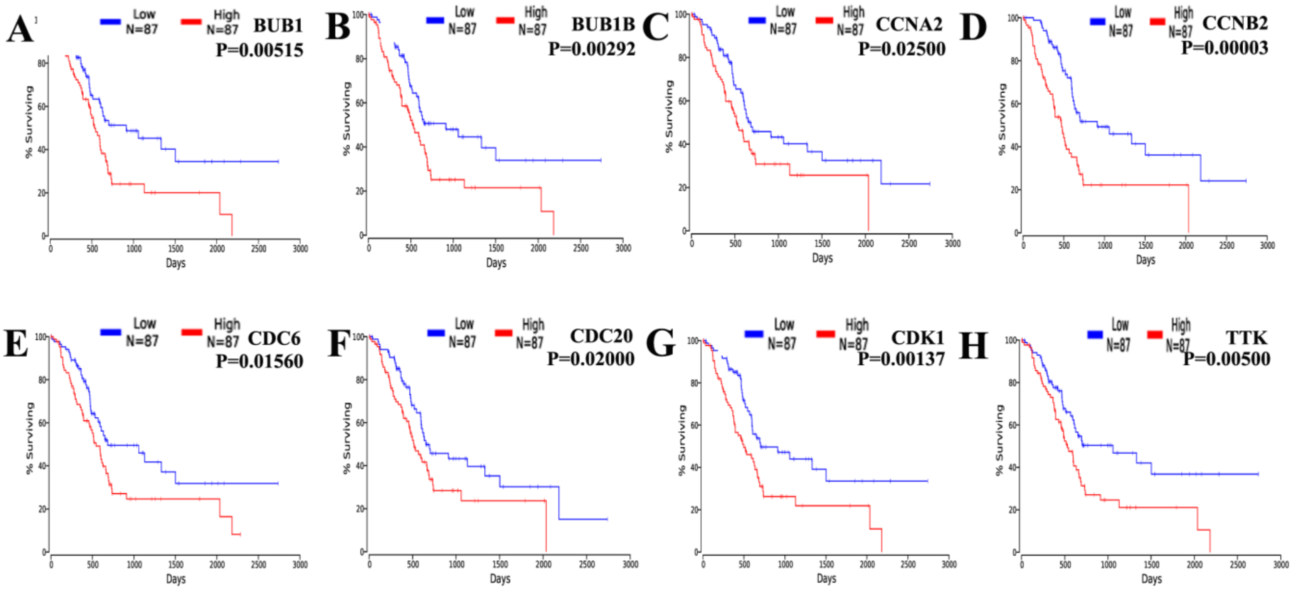

Supplementary Figure 1: The cell cycle-related genes in patients with PDAC. (A-H) separately showed the survival curve was negatively correlated with the prognosis in PDAC patients.

P<0.05 was considered to indicate a statistically significant

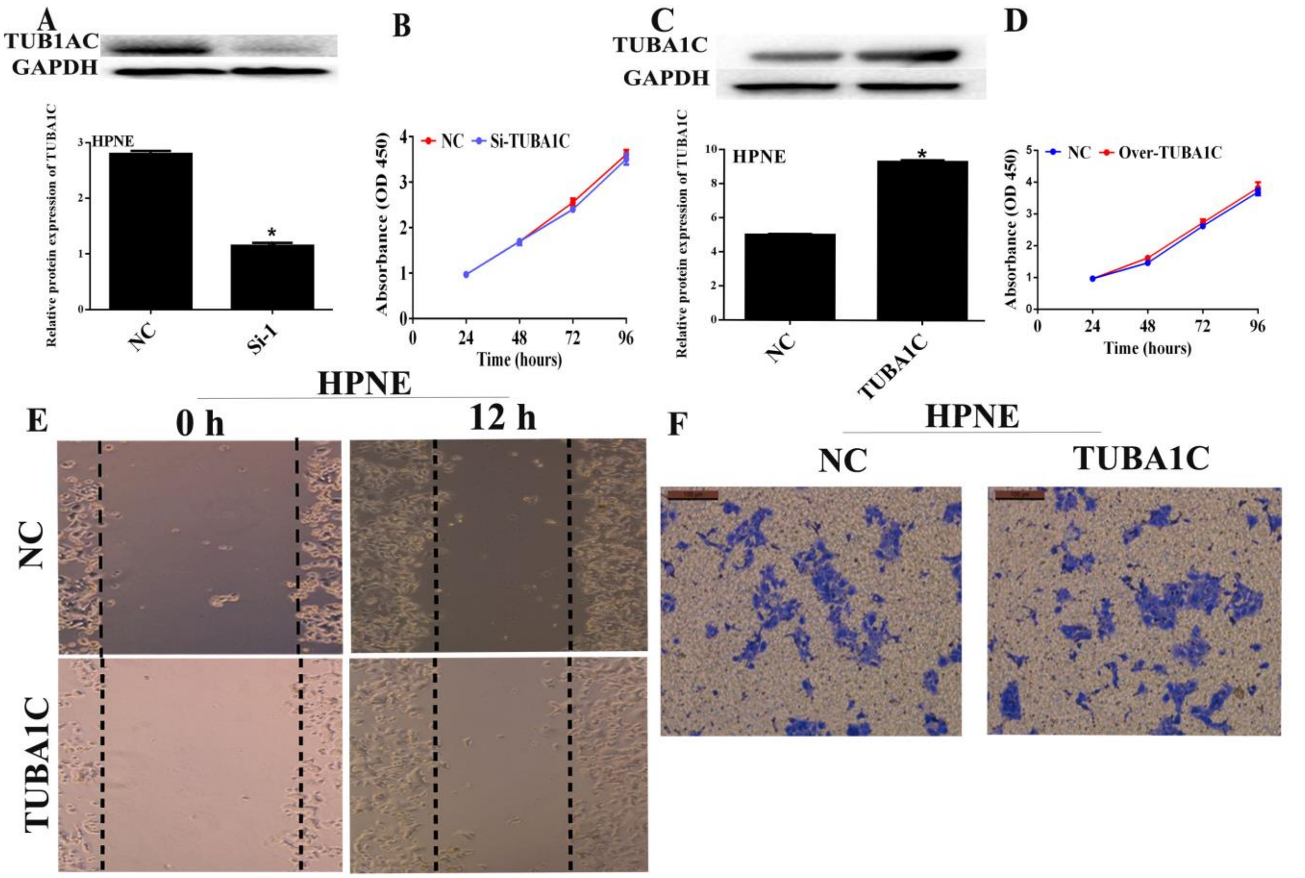

Supplementary Figure 2: knockdown or overexpression of TUBA1C had no effect on normal non-tumorigenic HPNE cells proliferation (A) Western blot analyses were performed to evaluate the knockdown of TUBA1C on HPNE cells；(B) CCK-8 assays showed that knockdown of TUBA1C does not affect the proliferation of HPNE cells (n = 3)；(C) Western blot analyses were performed to evaluate the TUBA1C overexpression on HPNE cells；(D) CCK-8 assays showed that TUBA1C overexpressing does not affect the proliferation of HPNE cells (n = 3)；

(E-F) wound healing and Transwell assays were performed to detect changes in migration and invasion of HPNE cells after TUBA1C overexpression (n = 3).

*P<0.05 was considered to indicate a statistically significant
